# Microwave-Assisted Desilication as a Route to Hierarchical Y Zeolites: Linking Pore Architecture, Acidity, and Catalytic Stability in VGO Cracking

**DOI:** 10.3390/molecules31152670

**Published:** 2026-07-31

**Authors:** Jayson Fals, Jhonnys D. Guerrero, Mayerlenis Jiménez Rojas, Nestor Cubillan, Edgar A. Márquez Brazón

**Affiliations:** 1 Grupo de Investigación en Oxi/Hidrotratamiento Catalítico y Nuevos Materiales, Programa de Química-Ciencias Básicas, Universidad del Atlántico, Barranquilla 080003, Colombia; 2 Grupo de Productos Naturales y Bioquímica de Macromoléculas, Facultad de Ciencias, Universidad del Atlántico, Puerto Colombia 081001, Colombia; 3 Grupo de Investigación en Biotecnología de Microalgas, Fisicoquímica Aplicada y Estudios Ambientales, Programa de Química, Universidad del Atlántico, Puerto Colombia 080003, Colombia; jguerrero@fiq.unl.edu.ar; 4 Instituto de Investigaciones en Catálisis y Petroquímica (INCAPE)/Consejo Nacional de Investigaciones Científicas y Técnicas (CONICET), Universidad Nacional del Litoral, Santa Fe CP 3000, Argentina; 5 Grupo Bioprocesos, Facultad de Ingeniería, Universidad de Antioquia, Medellín 050010, Colombia; mayerlenis.jimenez@udea.edu.co; 6 Programa de Química, Facultad de Ciencias Básicas, Universidad del Atlántico, Barranquilla 080003, Colombia; nestorcubillan@mail.uniatlantico.edu.co; 7 Departamento de Ciencias Naturales, Grupo de Investigación en Química y Biología, Facultad de Ciencias Básicas, Universidad del Norte, Barranquilla 080003, Colombia

**Keywords:** microwave-assisted desilication, hierarchical Y zeolites, fluid catalytic cracking, vacuum gas oil, Brønsted acidity, mesoporosity, catalyst deactivation, coke formation, nitrogen-containing compounds, catalytic stability

## Abstract

Hierarchical zeolites have emerged as an effective strategy to mitigate diffusional limitations and catalyst deactivation during the catalytic cracking of heavy feedstocks. However, conventional alkaline desilication often lacks selectivity, leading to partial loss of crystallinity and unfavorable alterations in acidic properties. In this work, microwave-assisted desilication is explored as an alternative route to engineer hierarchical Y zeolites with improved structural control and catalytic performance. A systematic comparison between conventional and microwave-assisted treatments was carried out using a 0.20 mol L^−1^ NaOH solution, followed by hydrothermal stabilization. The resulting materials were comprehensively characterized by X-ray diffraction, nitrogen physisorption, scanning electron microscopy, ICP–OES, and pyridine-adsorbed FTIR. Catalytic performance was evaluated in the cracking of nitrogen-containing vacuum gas oil under microactivity test conditions representative of FCC operation. Microwave-assisted desilication promotes a more homogeneous development of mesoporosity, yielding higher mesopore volumes and larger pore diameters while preserving a greater fraction of the FAU crystalline structure and Brønsted acidity compared to conventional treatment. These features translate into enhanced catalytic behavior, including higher and more stable conversions, increased gasoline selectivity (up to 63 wt%), and significantly reduced coke yields. In addition, spectroscopic and thermal analyses reveal that coke formed on the microwave-treated zeolite is less condensed and more readily oxidizable, indicating a reduced propensity for irreversible deactivation. Finally, the results demonstrate that the mode of energy input during desilication plays a critical role in dictating the balance between pore architecture and acidity, ultimately governing catalytic performance. Microwave-assisted desilication emerges as an efficient strategy for designing hierarchical Y zeolites with improved accessibility, selectivity, and resistance to deactivation under severe FCC conditions.

## 1. Introduction

Fluid catalytic cracking (FCC) remains one of the cornerstone technologies in modern petroleum refineries, enabling the conversion of heavy hydrocarbon fractions into lighter fuels and high-value products [1,2]. In these systems, Y zeolite constitutes the primary active phase due to its high density of Brønsted acid sites, well-defined microporous framework, and excellent hydrothermal stability. However, the processing of increasingly complex feedstocks such as vacuum gas oils (VGO), enriched in polyaromatic and nitrogen-containing compounds, imposes severe diffusional limitations within the FAU structure [3,4]. Restricted intracrystalline transport promotes prolonged residence times of reactive intermediates, favoring secondary reactions such as hydrogen transfer, dehydrogenation, and polycondensation, which ultimately lead to coke formation, pore blockage, and rapid catalyst deactivation [5,6,7,8].

To overcome these limitations, the development of hierarchical zeolites, combining intrinsic microporosity with an additional mesoporous network, has emerged as a robust strategy to enhance catalytic performance [9,10]. The presence of mesopores facilitates molecular transport, improves accessibility to active sites, and reduces the accumulation of carbonaceous species responsible for deactivation. Among the various approaches to generate hierarchical structures, alkaline desilication has gained considerable attention due to its simplicity, versatility, and industrial relevance. This method relies on the selective extraction of silicon from the framework, promoting the formation of intracrystalline mesoporosity [11,12]. Nevertheless, insufficient control of the desilication process may result in excessive silicon removal, loss of crystallinity, formation of extra-framework aluminum species, and undesirable modifications in acidity, highlighting the need for improved control over the treatment conditions [13].

Despite these advances, achieving an optimal balance between mesoporosity generation and preservation of structural and acidic integrity remains a central challenge in the design of FCC catalysts. In these systems, catalytic behavior is governed by the interplay between pore architecture, accessibility, and the distribution and strength of acid sites, which together determine activity, selectivity, and resistance to deactivation. In particular, coke formation—an inherent phenomenon in the cracking of heavy feedstocks—is strongly influenced by both diffusional constraints and acid site properties. Therefore, establishing clear relationships between structure, acidity, and catalytic performance is essential for developing more robust and efficient FCC catalysts capable of operating under increasingly severe conditions [14].

In this context, the mode of energy delivery during alkaline desilication has emerged as a key parameter influencing silicon extraction kinetics, framework preservation, and porous structure evolution. Conventional convective heating often leads to heterogeneous temperature distributions and limited control over desilication, whereas microwave-assisted heating provides rapid and volumetric energy transfer, resulting in more homogeneous thermal profiles and enhanced mass transport. The application of microwave irradiation in heterogeneous catalysis has demonstrated significant advantages, including reduced processing times, improved energy efficiency, and enhanced control over material properties, making it a promising route for process intensification in catalyst design [15].

Recent studies have shown that microwave-assisted desilication can accelerate the generation of intracrystalline mesoporosity while preserving the crystallinity of zeolites more effectively than conventional treatments. These improvements are attributed to the enhanced interaction between microwave radiation and the reaction medium, which promotes more uniform and selective silicon extraction. However, most available studies have focused on model zeolite systems, and the application of this strategy to commercial Y zeolites under FCC-relevant conditions remains comparatively limited. In particular, the impact of microwave-assisted desilication on the evolution of Brønsted and Lewis acidity, as well as its direct implications for catalytic behavior and coke formation during VGO cracking, has not been systematically established [16,17].

More importantly, the relationships between energy input mode, hierarchical pore development, acidity evolution, and catalyst deactivation remain insufficiently understood. In FCC processes, coke-induced deactivation arises from the progressive accumulation of carbonaceous species on acid sites and within the pore network, leading to loss of accessibility and catalytic activity. The nature, distribution, and reactivity of coke deposits are strongly governed by both structural and acidic properties of the catalyst, underscoring the importance of controlling these parameters during material design [18].

In this work, a systematic comparison between conventional and microwave-assisted alkaline desilication of a commercial Y zeolite is presented, with the objective of elucidating the relationships between desilication pathway, pore architecture, acidity, and catalytic performance. The resulting materials were comprehensively characterized in terms of their structural, textural, and acidic properties, and subsequently evaluated in the catalytic cracking of nitrogen-containing VGO under microactivity test conditions representative of FCC operation. By correlating physicochemical properties with catalytic performance, particularly conversion, product selectivity, and coke formation, this study provides new insights into the design of hierarchical Y zeolites with improved accessibility, enhanced catalytic stability, and greater resistance to deactivation under severe operating conditions.

## 2. Results and Discussion

### 2.1. VGO Properties

A comprehensive physicochemical characterization of the feedstocks was carried out in order to determine their main properties and to identify the presence of species that could influence catalytic performance during the catalytic cracking process. Table 1 summarizes the most relevant properties of the original vacuum gas oil (VGO), supplied by a Colombian refinery, and the doped feedstock (VGOD), which was prepared by the controlled addition of quinoline and indole to simulate heavy feedstocks rich in nitrogen-containing compounds. The original VGO exhibits an API gravity of 18.5, classifying it as a heavy petroleum fraction. This low API value is consistent with its high aromatic content (48.5 wt%), which increases the overall density of the feedstock and reflects a high degree of molecular complexity. The elevated aromaticity, particularly the presence of polyaromatic hydrocarbons, is a key factor governing catalytic cracking behavior, as it promotes condensation and dehydrogenation reactions that may lead to coke formation on the catalyst surface. The aniline point of the VGO (76.9 °C) and its refractive index (1.4930) further confirm the aromatic nature of the feedstock, in agreement with the SARA analysis, which shows comparable proportions of saturates (49.1 wt%) and aromatics (48.5 wt%), together with relatively low contents of resins (1.90 wt%) and asphaltenes (0.50 wt%). These low concentrations of heavy polar fractions suggest moderate colloidal stability and a reduced tendency toward pore blockage by large, highly polar species. The Conradson carbon residue (CCR) of the VGO is relatively low (0.46 wt%), indicating a limited intrinsic tendency toward coke formation under purely thermal conditions. However, under catalytic conditions, the high aromatic content may significantly contribute to coke generation through secondary reactions occurring on the acidic sites of the catalyst. The total nitrogen content of the VGO (0.10 wt%) lies within the typical range reported for vacuum gas oils, although it is sufficiently high to interfere with Brønsted acid sites through competitive adsorption. The concentrations of trace metals such as Ni, V, Na, and Fe are low in both feedstocks, indicating a minimal contribution of effects associated with metal-catalyzed dehydrogenation, coke promotion, or structural degradation of the zeolite framework. This allows the effects observed during catalytic testing to be attributed primarily to feedstock composition and catalyst properties rather than to metal poisoning phenomena. The simulated distillation curve further confirms the heavy nature of the VGO, with an initial boiling point of 270 °C, 95 vol% distilled below 529 °C, and a final boiling point of 575 °C. The wide boiling range reflects an extensive distribution of molecular weights and chemical structures, which represents an additional challenge for efficient conversion and favors the occurrence of secondary reactions associated with coke formation.

In order to specifically evaluate the impact of nitrogen-containing compounds on catalyst deactivation, the original VGO was subsequently doped with quinoline and indole, yielding the VGOD feedstock. Each compound was incorporated at a concentration of 0.40 wt%, resulting in a total nitrogen content of 0.90 wt%. As a consequence of the doping process, a slight decrease in API gravity (from 18.5 to 18.1), a reduction in the aniline point, an increase in the refractive index, and a shift in the distillation curve toward higher temperatures were observed, indicating a heavier feedstock with enhanced aromatic character. These changes are also reflected in the SARA distribution of the VGOD, where an increase in the aromatic fraction (51.1 wt%) and a slight rise in resin and asphaltene contents are observed. The simultaneous presence of quinoline and indole enables a realistic simulation of severe operating conditions, as both compounds exhibit distinct deactivation mechanisms: quinoline, due to its stronger basicity, tends to adsorb strongly on Brønsted acid sites, whereas indole, being a weaker base, may primarily contribute to physical pore blockage and diffusional limitations. Overall, the VGOD feedstock constitutes an appropriate model system for evaluating catalyst resistance to nitrogen-induced deactivation under FCC-relevant conditions.

### 2.2. Catalyst Properties

The structural evolution of the Y zeolites after alkaline desilication was investigated by X-ray diffraction (XRD) to assess changes in crystallinity and framework integrity associated with mesopore generation. In FAU-type zeolites, the formation of intracrystalline mesopores is typically reflected by a decrease in the intensity of the characteristic diffraction peaks, generally without significant shifts in peak position, indicating that the long-range structural order is largely preserved [19,20].

**Figure 1:**
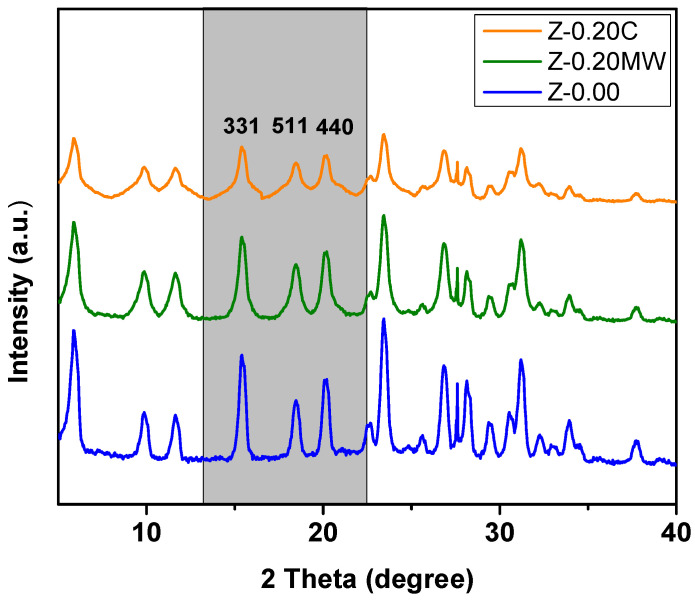
XRD patterns of parent and desilicated Y zeolites. The gray shaded area indicates the region containing the main diffraction peaks discussed in the text.

As shown in Figure 1, all samples (Z-0.00, Z-0.20C, and Z-0.20MW) exhibit the characteristic reflections of the FAU structure, confirming that the zeolitic framework is retained after both desilication treatments. The main diffraction peaks located at 2θ ≈ 15.7°, 18.7°, and 20.4°, corresponding to the (331), (511), and (440) crystallographic planes, respectively, remain clearly identifiable. However, a progressive decrease in peak intensity accompanied by moderate peak broadening is observed after treatment, indicating partial framework perturbation associated with silicon extraction.

This effect is quantitatively reflected in the crystallinity values (Table 2), where the conventional treatment results in a more pronounced loss (64%) compared to the microwave-assisted sample (79%). The stronger attenuation of diffraction peaks in Z-0.20C suggests a less selective alkaline attack and a higher degree of structural degradation [21]. In contrast, the sharper and more intense reflections observed for Z-0.20MW indicate that microwave-assisted desilication promotes a more controlled and homogeneous silicon extraction process, minimizing excessive framework damage while preserving the integrity of the FAU structure.

The textural properties of the zeolites were evaluated by nitrogen physisorption at 77 K, allowing a quantitative assessment of the modifications induced by alkaline desilication (Table 2). The parent material (Z-0.00) exhibits a typical microporous structure, with a high BET surface area (696 m^2^ g^−1^) and a micropore volume of 0.342 cm^3^ g^−1^, accounting for approximately 77% of the total pore volume, as expected for conventional FAU-type zeolites. The contribution of mesoporosity is limited, reflecting the intrinsic microporous nature of Y zeolite. Such pore structure is known to impose strong diffusional constraints when processing bulky hydrocarbon molecules in FCC reactions, highlighting the need for hierarchical modification [22].

After desilication, a clear redistribution of porosity is observed. In the conventionally treated sample (Z-0.20C), the micropore volume decreases significantly (0.342 → 0.234 cm^3^ g^−1^), accompanied by a reduction in BET surface area (696 → 584 m^2^ g^−1^), indicating partial loss of microporous structure and framework degradation. This loss is compensated by a substantial increase in mesopore volume (0.104 → 0.276 cm^3^ g^−1^) and enlargement of mesopore diameter (50.2 → 66.5 Å), confirming the generation of intracrystalline mesoporosity through silicon extraction. This behavior is consistent with the well-established desilication mechanism, where controlled removal of framework silicon leads to the formation of secondary porosity while partially disrupting the crystalline network.

In contrast, the microwave-assisted sample (Z-0.20MW) exhibits a more pronounced development of mesoporosity, reaching a mesopore volume of 0.348 cm^3^ g^−1^ and an average pore diameter of 88.4 Å. Notably, this is achieved while maintaining a relatively high BET surface area (625 m^2^ g^−1^), significantly higher than that of Z-0.20C. This indicates that microwave-assisted desilication promotes a more selective and homogeneous silicon extraction process, minimizing excessive micropore collapse while enabling the formation of larger and more accessible mesopores. Such behavior has been attributed to the volumetric and rapid heating associated with microwave irradiation, which enhances mass transfer and improves control over desilication kinetics. Consequently, Z-0.20MW exhibits a more favorable balance between mesoporosity generation and micropore preservation, which is crucial to maximize molecular transport without severely compromising active site density.

The framework Si/Al ratios, calculated according to the Breck–Flanigen correlation, further support the structural modifications induced by alkaline treatment. Both desilicated samples exhibited lower framework Si/Al ratios than the parent zeolite, confirming the extraction of framework silicon during desilication. Notably, the microwave-treated sample retained a slightly higher framework Si/Al ratio than the conventionally treated zeolite, suggesting a more controlled silicon removal, in agreement with its higher crystallinity and better preservation of Brønsted acidity.

The evolution of acidic properties (Table 2), determined by pyridine adsorption FTIR, reflects the structural modifications induced by desilication. The parent zeolite (Z-0.00) shows the highest Brønsted acidity (186 μmol Py g^−1^), consistent with the high density of framework Si–OH–Al bridges in the intact FAU structure. Upon desilication, Brønsted acidity decreases due to partial removal and redistribution of framework aluminum associated with silicon extraction, a phenomenon widely reported during alkaline treatment of zeolites [23].

The conventionally treated sample (Z-0.20C) shows a pronounced reduction in Brønsted acidity (186 → 158 μmol Py g^−1^), consistent with its lower crystallinity and greater structural perturbation. In contrast, the microwave-assisted sample (Z-0.20MW) retains a higher Brønsted acidity (172 μmol Py g^−1^), in agreement with its improved structural preservation. This indicates that microwave-assisted desilication limits excessive framework disruption and better preserves catalytically active protonic sites. From a catalytic perspective, this preservation is essential, as Brønsted acid sites govern carbocation-mediated cracking pathways in FCC reactions [24].

Simultaneously, desilication leads to an increase in Lewis acidity, associated with the formation of extra-framework aluminum species. This effect is more pronounced in Z-0.20C (82 μmol Py g^−1^), suggesting a higher degree of defect generation and framework collapse. The microwave-treated sample exhibits a lower density of Lewis sites (74 μmol Py g^−1^), indicating a more controlled desilication process. This distinction is particularly relevant, as excessive Lewis acidity is often associated with non-selective reactions and enhanced coke formation in zeolite catalysts. Therefore, Z-0.20MW achieves a more favorable acid site distribution, combining sufficient Brønsted acidity with limited defect formation, which is expected to enhance catalytic selectivity and stability. The results presented in Table 2 demonstrate that the desilication strategy critically governs the interplay between pore architecture and acidity. While conventional treatment enhances mesoporosity at the expense of structural integrity and Brønsted acid site density, microwave-assisted desilication enables the simultaneous development of a highly accessible mesoporous network and the preservation of a significant fraction of active sites. This optimized balance is essential for catalytic applications involving bulky molecules, as it enhances molecular diffusion, reduces residence time of reactive intermediates, and suppresses secondary reactions leading to coke formation [25]. The improved structure–acidity synergy observed for Z-0.20MW therefore provides a strong physicochemical basis for its enhanced catalytic performance during VGO cracking.

**Figure 2:**
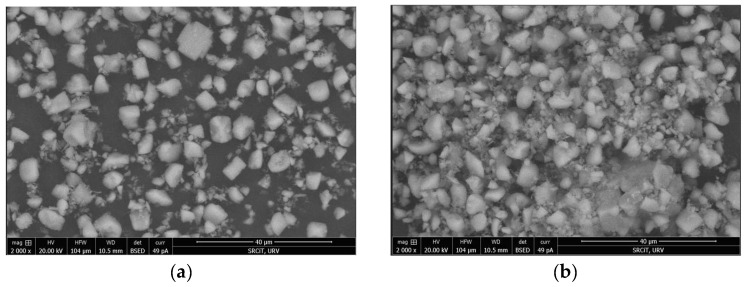
SEM micrographs of Y zeolites desilicated by microwave-assisted alkaline treatment (**a**) and conventional alkaline treatment (**b**).

The morphological differences observed by SEM (Figure 2) have direct implications for catalytic performance during VGO cracking. In the microwave-assisted sample (Z-0.20MW), the preservation of well-defined crystal facets and the lower degree of agglomeration indicate a more uniform external surface and improved particle dispersion. These features enhance external mass transfer and facilitate the access of bulky hydrocarbon molecules to the hierarchical pore network. Consequently, the improved accessibility reduces diffusion limitations and shortens the residence time of reactive intermediates, suppressing secondary reactions responsible for overcracking and coke formation. This relationship between morphology, accessibility, and catalytic efficiency has been widely recognized in hierarchical zeolites, where enhanced transport properties directly translate into improved activity and selectivity [26].

In contrast, the conventionally treated sample (Z-0.20C) exhibits rough surfaces, particle aggregation, and the presence of surface debris, which can partially block pore entrances and create local diffusion barriers. These morphological defects increase mass-transfer resistance and promote longer residence times of intermediate species within the catalyst. Under such conditions, secondary reactions such as hydrogen transfer, aromatization, and polycondensation become more favorable, leading to increased formation of light gases and coke deposits. The role of diffusional constraints and surface heterogeneities in promoting coke formation and non-selective pathways is well established in FCC catalysis and is strongly associated with deactivation phenomena [26]. Moreover, the dissolution–reprecipitation phenomena inferred for Z20C may generate heterogeneous external surfaces with a non-uniform distribution of active sites, contributing to localized acidity variations and less controlled reaction pathways. In contrast, the more homogeneous morphology of Z-0.20MW suggests a more uniform distribution of accessible acid sites, which is essential for promoting controlled cracking reactions. The combination of structural uniformity and balanced accessibility is known to favor selective pathways toward gasoline-range hydrocarbons while limiting excessive secondary transformations [27].

Finally, the SEM observations provide direct morphological evidence that complements the structural (XRD), textural (physisorption), and acidic (Py-FTIR) analyses discussed previously. The superior preservation of crystal integrity and reduced aggregation in the microwave-treated zeolite enhance molecular transport and accessibility, which ultimately translates into higher catalytic stability, improved gasoline selectivity, and lower coke formation during VGO cracking. These results highlight that, beyond pore structure and acidity, external morphology plays a critical role in governing catalytic performance in hierarchical zeolites.

### 2.3. Catalytic Activity

The catalytic activity of the zeolites was evaluated through catalytic cracking tests using vacuum gas oil doped with nitrogen-containing compounds as a model feedstock, in order to simulate a representative heavy feed and to compare the intrinsic activity of the zeolites, as well as their performance in terms of conversion and product selectivity. The experiments were carried out in a MAT-type reactor under controlled conditions, so that the observed differences could be mainly attributed to the structural, textural, and acidic modifications induced by the desilication treatments. In particular, the effect of conventional and microwave-assisted desilication methods on the development of hierarchical mesoporosity, the preservation of Brønsted acidity, and their impact on VGOD accessibility and cracking efficiency was analyzed. In addition, special attention was paid to catalyst deactivation phenomena, evaluating the influence of coke deposition (nature of coke) and nitrogen compounds on the acidic and structural properties of the catalysts.

#### 2.3.1. Conversion and Product Distribution

**Figure 3:**
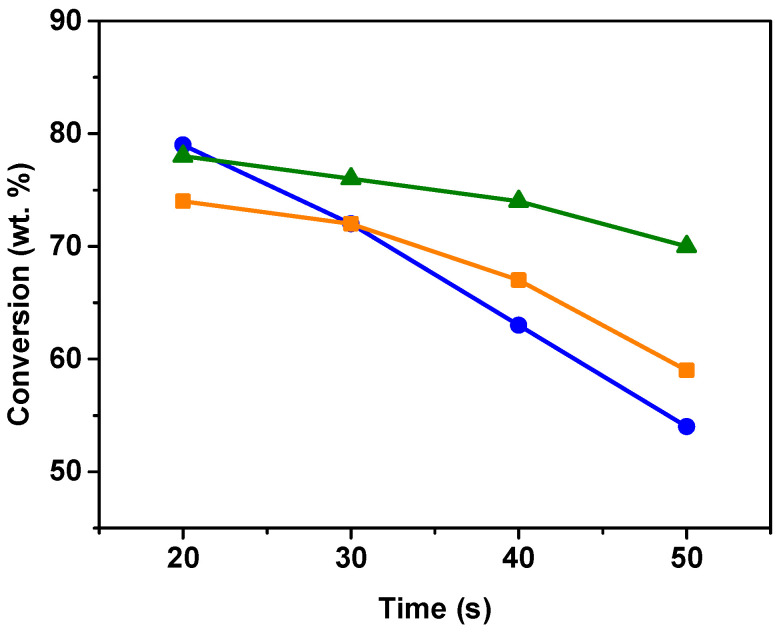
VGOD conversion as a function of reaction time at 550 °C on Z-0.00 (●), Z-0.20C (**□**) and Z-0.20MW (▲).

Figure 3 shows the evolution of VGOD conversion as a function of reaction time over the parent and desilicated Y zeolites. As observed in Figure 3, conversion decreases progressively with increasing contact time (20–50 s) for all samples, which is indicative of cumulative catalyst deactivation during the reaction. However, significant differences are evident among the catalysts, reflecting the strong influence of structural, textural, and acidic properties on catalytic stability. The microwave-assisted zeolite (Z-0.20MW) consistently exhibits the highest conversions and the smallest decline with increasing reaction time, whereas the parent zeolite (Z-0.00) shows the most pronounced deactivation. The conventionally treated sample (Z-0.20C) displays intermediate behavior. These trends clearly demonstrate that the introduction of hierarchical porosity improves resistance to deactivation, with a more pronounced effect for the microwave-assisted treatment.

At short reaction times (20 s), all samples exhibit relatively high conversions (≈78–79 wt%), with the parent zeolite (Z-0.00) showing slightly higher initial activity. This behavior can be attributed to its higher crystallinity and greater density of strong Brønsted acid sites (40%), which favor rapid C–C bond cleavage through carbocationic mechanisms during the initial stages of cracking. However, as reaction time increases, Z-0.00 experiences a sharp decline in conversion (from ~79 wt% to ~54 wt% at 50 s). This pronounced deactivation is associated with severe diffusional limitations resulting from its predominantly microporous nature (Vmicro = 0.342 cm^3^ g^−1^), which promotes the accumulation of bulky aromatic intermediates and nitrogen-containing species within the pore network, leading to acid site neutralization and pore blockage.

The conventionally desilicated zeolite (Z-0.20C) exhibits a lower initial conversion, consistent with its reduced crystallinity (64%) and lower Brønsted acidity (158 μmol Py g^−1^). Nevertheless, the decline in conversion with reaction time is less pronounced compared to Z-0.00. This improved stability can be attributed to the increase in mesopore volume (0.276 cm^3^ g^−1^) and pore diameter (66.5 Å), which enhance molecular diffusion and alleviate intracrystalline transport limitations. However, despite these improvements, the performance of Z-0.20C remains limited by partial structural degradation and the higher density of Lewis acid sites, which are associated with non-selective reactions and coke formation.

In contrast, the microwave-assisted zeolite (Z-0.20MW) exhibits the most favorable catalytic behavior over the entire time range. This sample combines high initial conversion (~78 wt%) with a significantly smaller decrease in activity (~70 wt% at 50 s), demonstrating superior resistance to deactivation. This performance can be rationalized by its optimized physicochemical properties: a highly developed mesoporous network (Vmeso = 0.348 cm^3^ g^−1^; dp = 88.4 Å), relatively high crystallinity (79%), and better preservation of Brønsted acidity (172 μmol Py g^−1^). The improved accessibility provided by the hierarchical pore structure facilitates the diffusion of bulky molecules and nitrogen compounds, reduces their residence time within the zeolite, and limits the probability of secondary reactions leading to coke formation.

The progressive decrease in conversion observed for all catalysts at longer reaction times is therefore the result of a complex interplay between coke deposition and nitrogen-induced deactivation. However, the extent of this deactivation is strongly governed by the balance between acidity and pore accessibility. In this context, Z-0.20MW exhibits the optimal compromise, where sufficient Brønsted acidity ensures high intrinsic activity while enhanced mesoporosity mitigates diffusion limitations and coke accumulation. These results clearly demonstrate that catalytic stability under FCC conditions is not dictated solely by acid site density, but rather by the synergistic interaction between pore architecture, acidity, and resistance to deactivation.

Beyond the differences observed in conversion, the distribution of products is strongly influenced by both catalyst properties and reaction conditions, as summarized in Table 3. With increasing contact time, all catalysts exhibit a general increase in gas and coke selectivities, accompanied by a decline in gasoline yield. This trend reflects the increasing severity of cracking conditions and the progressive contribution of secondary reactions, including overcracking, hydrogen transfer, and condensation processes, which are known to become more dominant as catalyst deactivation proceeds. However, the magnitude of these changes varies significantly depending on the catalyst structure.

The parent zeolite (Z-0.00) exhibits the least favorable product distribution, with the lowest gasoline selectivity (52.0 wt%) and the highest yields of dry gas (16.2 wt%) and coke (12.8 wt%). This behavior can be directly attributed to its predominantly microporous structure and high fraction of strong Brønsted acid sites (40%), which promote extensive secondary cracking and hydrogen-transfer reactions. The restricted diffusion within the micropores (V_micro = 0.342 cm^3^ g^−1^) enhances the residence time of reactive intermediates, leading to overcracking of primary products and facilitating the formation of coke precursors. As a result, valuable gasoline-range products are further degraded into lighter gases or transformed into carbonaceous deposits.

The conventionally desilicated sample (Z-0.20C) exhibits a more balanced product distribution, with increased gasoline selectivity (58.5 wt%) and reduced coke formation (9.5 wt%). As shown in Table 3, the decrease in dry gas yield (16.2 → 11.6 wt%) and the increase in gasoline fraction indicate a partial suppression of secondary cracking reactions. These improvements can be linked to the development of mesoporosity (V_meso = 0.276 cm^3^ g^−1^), which enhances molecular diffusion and reduces the residence time of intermediate species. However, the presence of a higher density of Lewis acid sites (82 μmol Py g^−1^), associated with extra-framework aluminum species, suggests that non-selective pathways are still promoted, limiting the overall performance.

In contrast, the microwave-assisted zeolite (Z-0.20MW) exhibits the most favorable product distribution, achieving the highest gasoline selectivity (63.0 wt%), lowest dry gas yield (7.10 wt%), and lowest coke formation (6.6 wt%). These results clearly demonstrate that the optimized combination of hierarchical porosity and preserved Brønsted acidity effectively suppresses secondary reactions. The larger mesopore volume (0.348 cm^3^ g^−1^) and pore diameter (88.4 Å) facilitate rapid diffusion of both reactants and primary products, minimizing their further transformation into undesirable products.

The analysis of gasoline composition further reinforces this interpretation. As shown in Table 3, the gasoline fraction obtained over Z-0.20MW is enriched in olefins (35.5%) and aromatics (30.2%), while paraffinic content is reduced (16.0%), compared to Z-0.00. This shift indicates a more favorable reaction network in which primary cracking and mild secondary reactions are promoted without excessive overconversion. Consequently, the research octane number (RON) increases from 84.0 (Z-0.00) to 89.7 (Z-0.20MW), highlighting a significant improvement in fuel quality.

These results demonstrate that product distribution is controlled by a delicate balance between acidity and accessibility. While high Brønsted acidity promotes initial cracking activity, excessive confinement within micropores enhances secondary reactions that reduce gasoline yield and increase coke formation. The microwave-assisted zeolite achieves an optimal compromise, where sufficient acidity is retained while hierarchical porosity improves diffusion and limits undesired transformations. This synergy between pore structure and acidity, previously evidenced by XRD, physisorption, and SEM analyses, translates directly into superior catalytic selectivity and product quality under FCC-relevant conditions.

##### Coke Yield

**Figure 4:**
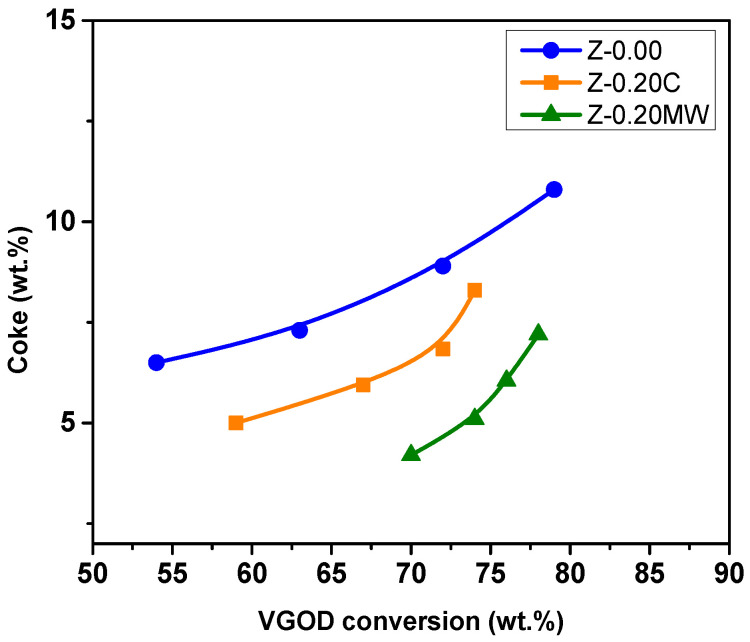
Coke yield over parent and desilicated Y zeolites: Z-0.00 (●), Z-0.20C (**□**), and Z-0.20MW (▲).

Figure 4 presents the evolution of coke yield as a function of VGOD conversion over the evaluated Y zeolite catalysts. A consistent increase in coke formation with conversion is observed for all samples, which is a well-documented trend in the catalytic cracking of heavy feedstocks enriched in polyaromatic and nitrogen-containing compounds. This behavior is typically associated with the progressive transformation of reactive intermediates through hydrogen-transfer, dehydrogenation, cyclization, and condensation reactions, ultimately leading to the formation of polyaromatic coke precursors and carbonaceous deposits. These mechanistic pathways over acid catalysts have been extensively described in the literature on zeolite catalysis and coke chemistry [28,29].

Despite this general trend, noticeable differences among the catalysts are evident, underscoring the key role of porosity and acidity in controlling coke formation. The parent zeolite (Z-0.00) exhibits the highest coke yields across the entire conversion range. This behavior can be attributed to its predominantly microporous structure and high density of strong Brønsted acid sites. The confined pore network of the FAU framework imposes significant diffusional limitations, increasing the residence time of bulky intermediates within the micropores and promoting their transformation into polyaromatic coke species. Such diffusion-controlled coke formation and steric trapping effects are well established for microporous zeolites [30].

In contrast, both desilicated zeolites show a clear reduction in coke yield at comparable conversion levels, confirming the beneficial effect of introducing hierarchical porosity. The conventionally treated sample (Z-0.20C) exhibits intermediate coke yields, which can be associated with the increased mesopore volume and improved accessibility to active sites. The presence of mesopores facilitates the diffusion of bulky molecules and enhances the removal of reaction intermediates from the catalyst interior, thus limiting their participation in secondary condensation reactions. The advantages of hierarchical zeolites in enhancing mass transport and catalyst efficiency are widely documented [31].

However, the benefits of improved transport in Z-0.20C appear to be partially offset by structural degradation induced during alkaline treatment. The generation of extra-framework aluminum species and the increased presence of Lewis acid sites can promote non-selective pathways, including deep dehydrogenation and condensation reactions, which sustain coke formation. These effects are consistent with reported consequences of uncontrolled desilication and framework damage in zeolites.

The microwave-assisted desilicated zeolite (Z-0.20MW) delivers the lowest coke yields across the studied conversion range, demonstrating superior resistance to coke deposition. This enhanced performance can be attributed to its optimized hierarchical structure, combining a well-developed mesoporous network with a high degree of crystallinity and preserved Brønsted acidity. This balance promotes efficient diffusion and reduces the residence time of reactive intermediates, thereby suppressing secondary reactions responsible for coke formation. The relationship between hierarchical porosity, improved accessibility, and reduced coke formation has been consistently reported for advanced zeolite catalysts [32,33].

This interpretation is further supported by spectroscopic and oxidation analyses, which typically show that improved mass transport reduces the formation of highly condensed aromatic coke species. In hierarchical systems, coke deposits tend to be less polyaromatic and more easily oxidizable, reflecting the reduced extent of secondary condensation reactions. These trends are consistent with established descriptions of coke structure and evolution in zeolite-based catalytic systems [34].

### 2.4. Coke Nature

To gain further insight into the deactivation phenomena occurring during VGOD catalytic cracking, the nature of the carbonaceous deposits formed on the zeolites was examined by FTIR spectroscopy. Particular attention was given to the absorption bands located around 1580 and 1610 cm^−1^, which are commonly used to differentiate between distinct families of coke species. The band near 1580 cm^−1^ is generally assigned to C = C stretching vibrations in condensed polyaromatic structures, which are characteristic of highly evolved and refractory coke. In contrast, the signal around 1610 cm^−1^ is typically associated with less condensed olefinic and alkyl-aromatic species that retain a higher H/C ratio and lower structural complexity. Therefore, the relative intensities of these bands provide valuable qualitative information about the degree of aromatic condensation and the evolution stage of the coke [35].

**Figure 5:**
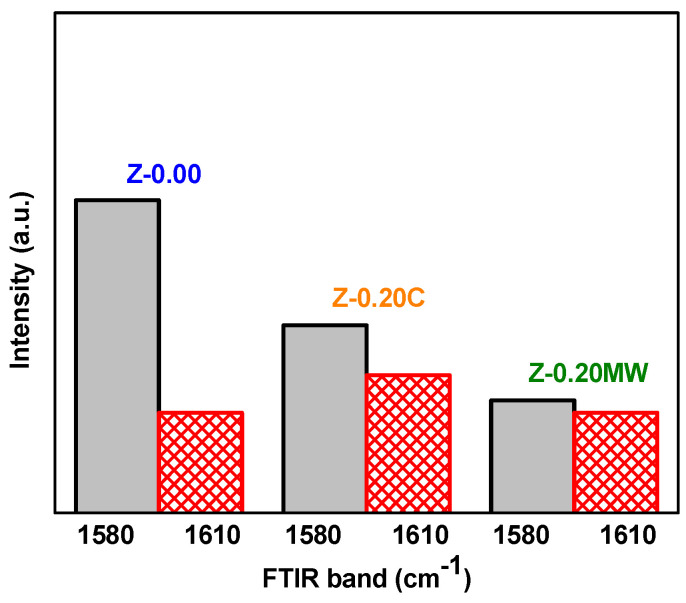
Intensities of the FTIR bands assigned to aromatic coke (1580 cm^−1^) and olefinic coke (1610 cm^−1^) formed in the conversion of VGOD at 550 °C (72 wt% conversion). Gray bars correspond to the FTIR band at 1580 cm^−1^, whereas red hatched bars correspond to the FTIR band at 1580 cm^−1^.

As shown in Figure 5, all catalysts exhibit contributions from both types of species, although their relative proportions differ significantly depending on catalyst properties. The parent zeolite (Z-0.00) shows a clear predominance of the band at 1580 cm^−1^, indicating a strong tendency toward the formation of highly condensed aromatic coke. This observation is in line with its higher coke yields and more pronounced deactivation behavior. The formation of such refractory deposits can be rationalized by the intrinsic characteristics of the material, namely its high density of strong Brønsted acid sites and its purely microporous FAU structure. These features favor the retention of bulky reaction intermediates within the pore network, increasing their residence time and promoting a sequence of hydrogen-transfer, cyclization, and dehydrogenation reactions that gradually lead to polyaromatic structures with low H/C ratios. In addition, the restricted diffusional environment hinders the desorption of these species, further enhancing their evolution toward more stable and condensed coke deposits [36,37].

The introduction of mesoporosity through desilication clearly alters this behavior. In the case of the conventionally treated sample (Z-0.20C), a decrease in the relative intensity of the 1580 cm^−1^ band is observed, suggesting a partial suppression of highly condensed aromatic coke formation. This can be attributed to the improved accessibility and reduced diffusional resistance associated with the presence of mesopores, which facilitate the transport of bulky molecules and shorten their residence time within the catalyst. Nevertheless, a significant contribution of aromatic coke is still detected, indicating that secondary condensation reactions are not completely suppressed. This behavior can be linked to the structural modifications induced during alkaline treatment, including partial loss of crystallinity and the formation of extra-framework aluminum species. These changes increase the contribution of Lewis acidity and structural heterogeneity, which are known to favor non-selective pathways such as deep dehydrogenation and aromatization, ultimately promoting coke condensation [38].

A more pronounced shift in coke composition is observed for the microwave-assisted desilicated zeolite (Z-0.20MW). In this case, the relative intensity of the 1580 cm^−1^ band is significantly reduced, while the contribution of the band at 1610 cm^−1^ becomes more prominent, indicating the formation of less condensed carbonaceous species. This spectral signature suggests that coke deposits remain at earlier stages of evolution, consisting predominantly of olefinic and alkyl-aromatic structures with higher hydrogen content. Such behavior is consistent with the improved catalytic stability and lower coke yields observed for this material. The origin of this effect can be directly associated with its optimized hierarchical architecture, which combines enhanced mesoporosity with good preservation of the microporous framework and Brønsted acidity. This balance promotes efficient molecular transport, allowing rapid diffusion of intermediates and limiting the extent of secondary reactions responsible for coke maturation.

From a practical standpoint, these differences in coke composition are highly relevant. Less condensed carbonaceous species, such as those predominant in Z-0.20MW, are generally more reactive toward oxidation and can be removed under milder regeneration conditions. In contrast, highly condensed polyaromatic coke, such as that formed on the parent zeolite, is more refractory and requires harsher regeneration, which can accelerate catalyst degradation. Therefore, beyond reducing the overall coke amount, the microwave-assisted desilication approach also improves coke quality by shifting its composition toward more easily oxidizable species.

Overall, the FTIR results provide clear evidence that the introduction of hierarchical porosity not only reduces coke formation but also modifies its chemical nature. In particular, microwave-assisted desilication promotes the formation of less evolved carbonaceous deposits with a lower degree of aromatic condensation. This behavior reflects an improved balance between accessibility, acidity, and structural integrity, which limits coke maturation pathways. From an FCC perspective, this dual effect—lower coke yield and enhanced coke reactivity—translates into improved catalyst stability, reduced regeneration severity, and enhanced process efficiency under heavy feed cracking conditions.

**Figure 6:**
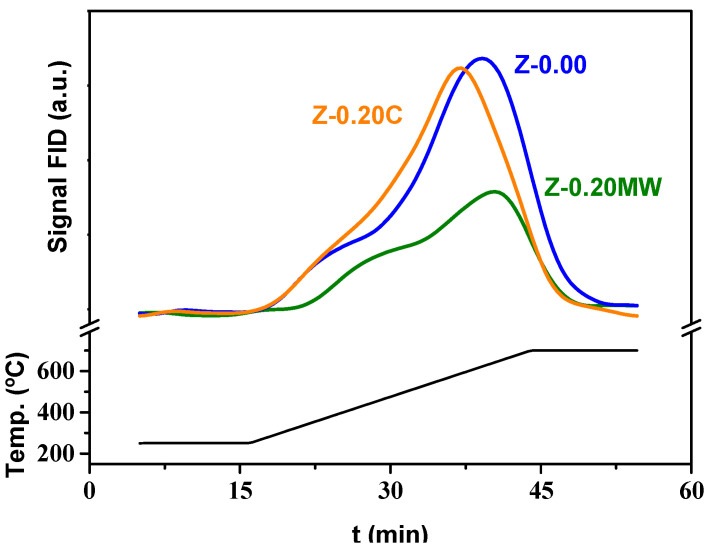
Temperature-programmed oxidation (TPO) profiles of coke deposits formed during VGOD cracking over Y zeolites.

Figure 6 presents the TPO profiles of the coke formed on the zeolites after VGOD cracking, providing complementary information to the FTIR analysis discussed previously. In all cases, the main combustion events occur above 500 °C, which is indicative of the predominance of highly condensed carbonaceous species with significant thermal stability. This observation is consistent with the FTIR results, where all samples exhibited contributions from the band at ~1580 cm^−1^ associated with polyaromatic structures. From a mechanistic standpoint, these species originate from successive hydrogen-transfer, cyclization, and dehydrogenation reactions typical of the carbocationic cracking of heavy feeds, leading to the gradual evolution of coke toward more condensed and refractory forms.

Despite this general behavior, the TPO profiles clearly reveal differences in both combustion temperature and peak shape, reflecting variations in coke composition, location, and accessibility. These differences follow the same order already identified by FTIR, reinforcing the close relationship between coke nature and oxidation behavior. In particular, the parent zeolite (Z-0.00) exhibits the most intense signal in the high-temperature region, with a combustion maximum shifted toward higher temperatures compared to the desilicated samples. This feature confirms that coke deposited on Z-0.00 is more refractory, in agreement with the higher contribution of condensed aromatic species detected by FTIR. The microporous FAU structure, combined with a high density of strong Brønsted acid sites, promotes the confinement of bulky intermediates and extends their residence time, favoring their progressive transformation into highly condensed polyaromatic structures. As a result, the coke formed is both structurally more evolved and more resistant to oxidation.

The broadness of the combustion profile observed for Z-0.00 provides additional insight into the spatial distribution of the deposited carbon. The presence of a high-temperature tail suggests that a significant fraction of the coke is located within the internal microporous network, where diffusion of oxygen is restricted during oxidation. Coke trapped in supercages and channel intersections experiences stronger confinement and limited accessibility, leading to delayed combustion at higher temperatures. This behavior is fully consistent with the severe diffusional constraints of the parent zeolite and reinforces the idea that catalyst deactivation is not only driven by coke quantity, but also by its location within the porous structure.

The desilicated sample prepared by conventional treatment (Z-0.20C) shows a slight shift in the oxidation profile toward lower temperatures, indicating a partial reduction in coke condensation. This trend aligns well with the decrease in the relative intensity of the 1580 cm^−1^ band observed by FTIR, confirming that mesoporosity mitigates, although does not fully suppress, the formation of highly aromatic coke. The introduction of mesopores improves molecular transport and facilitates the removal of intermediates before extensive polycondensation occurs. However, the persistence of a significant high-temperature contribution in the TPO profile indicates that refractory coke species are still formed. This behavior can be associated with the structural perturbations induced during alkaline desilication. The presence of extra-framework aluminum and defect sites creates heterogeneous environments that favor non-selective dehydrogenation and aromatization pathways, thereby sustaining the formation of condensed carbonaceous structures. Consequently, Z-0.20C displays an intermediate behavior, where improved diffusion coexists with structural features that still promote coke maturation.

A markedly different oxidation pattern is observed for the microwave-assisted desilicated zeolite (Z-0.20MW). In this case, the combustion profile is clearly shifted toward lower temperatures and the intensity of the high-temperature region is significantly reduced. This result directly supports the FTIR findings, where a higher contribution of the band at ~1610 cm^−1^ indicated the predominance of less condensed coke species. The lower oxidation temperature reflects the formation of carbonaceous deposits with higher H/C ratios, lower aromaticity, and reduced structural order. These species are typically considered “softer” coke and are more readily oxidized.

The origin of this behavior can be traced back to the optimized hierarchical structure of Z-0.20MW. The combination of enhanced mesoporosity with a well-preserved FAU framework facilitates rapid diffusion of bulky VGOD molecules and efficient removal of intermediates from the catalyst interior. As a result, the residence time of reactive species is significantly shortened, suppressing the sequence of reactions leading from olefinic intermediates to fully condensed polyaromatic coke. In addition, the lower concentration of extra-framework aluminum species limits the formation of strong Lewis acid sites that are often associated with deep dehydrogenation and non-selective condensation pathways. This balance between accessibility and acidity effectively hinders the evolution of coke toward highly stable and refractory structures.

From an operational perspective, the differences evidenced by TPO are particularly relevant. The lower combustion temperatures observed for Z-0.20MW indicate that coke deposited on this catalyst can be removed under milder regeneration conditions. In contrast, the high-temperature oxidation required for Z-0.00 reflects the presence of more stable and less accessible coke, which demands harsher regeneration and can accelerate catalyst degradation. Therefore, the TPO results not only confirm the trends observed by FTIR, but also provide direct evidence of improved coke manageability in hierarchical systems.

Overall, the combined FTIR and TPO analyses offer a consistent picture of coke formation and evolution across the studied catalysts. The parent zeolite promotes the formation of highly condensed, micropore-confined coke that is difficult to oxidize and strongly contributes to deactivation. Conventional desilication partially alleviates these effects but introduces structural features that still favor coke maturation. In contrast, microwave-assisted desilication effectively modifies both the formation pathways and final structure of carbonaceous deposits, leading to less condensed, more accessible, and more easily oxidizable coke. This dual effect—reduced coke severity and improved regenerability—underpins the enhanced stability of Z-0.20MW under demanding VGOD cracking conditions and highlights the importance of controlling both porosity and acidity in FCC catalyst design.

The TPO profiles obtained in this work are fully consistent with the general behavior reported for FCC catalysts processing heavy feeds, where coke oxidation typically occurs at temperatures above 500 °C due to the predominance of polyaromatic structures. Similar high-temperature combustion features have been widely described for coke formed through sequential hydrogen-transfer, cyclization, and dehydrogenation reactions over acidic zeolites, which progressively generate highly condensed and thermally stable carbon deposits. In this context, the profiles observed for the parent Z-0.00 sample closely resemble those reported for conventional catalysts under diffusion-limited conditions, where coke evolves toward polycyclic aromatic structures strongly bound within the microporous network [39].

The high combustion temperature and broad oxidation profile observed for Z-0.00 further indicate a heterogeneous distribution of coke species located both on the external surface and deep within the micropores. This behavior has been extensively reported in the literature, where coke trapped in FAU supercages and channels exhibits delayed oxidation due to restricted oxygen diffusion and strong steric confinement. Such “hard coke” is typically associated with severe catalyst deactivation, as it blocks access to active sites and requires harsh regeneration conditions. Therefore, the results for Z-0.00 are in line with classical descriptions of coke location and reactivity in purely microporous zeolite systems.

When comparing the desilicated samples with literature reports on hierarchical zeolites, a clear shift toward lower oxidation temperatures is observed, indicating a decrease in coke condensation. This trend has been consistently attributed to the introduction of mesoporosity, which enhances molecular transport and limits the residence time of reactive intermediates within the zeolite framework. Previous studies on desilicated Y and ZSM-5 zeolites have shown similar behavior, where improved accessibility reduces the extent of secondary reactions leading to polyaromatic coke. The intermediate behavior of Z-0.20C agrees well with these findings, confirming that conventional alkaline desilication partially alleviates diffusional limitations but does not fully prevent coke maturation [40].

However, an important distinction emerges when comparing your results with conventional hierarchical zeolites reported in the literature. While many studies highlight the benefits of desilication, they also point out the detrimental effects associated with framework degradation, including the formation of extra-framework aluminum species and increased Lewis acidity, which can promote unselective dehydrogenation and aromatization pathways. The relatively high-temperature contribution still observed for Z-0.20C is fully consistent with these reports, indicating that structural defects can counterbalance the advantages of improved diffusion. This reinforces the idea that not all hierarchical modifications are equally effective, and that preserving structural integrity is a key factor governing coke evolution [41].

In contrast, the behavior of Z-0.20MW clearly deviates from what is typically reported for conventionally treated hierarchical zeolites and highlights the specific advantages of microwave-assisted desilication. The pronounced shift in the TPO profile, together with the reduced intensity of the high-temperature region, indicates the formation of coke that is less condensed. Similar trends have been observed in advanced hierarchical systems with optimized pore connectivity, where improved mass transport suppresses the transformation of intermediate species into highly polyaromatic coke. However, the magnitude of the shift observed in Z-0.20MW suggests a more effective control over coke evolution, which can be attributed to the more uniform and controlled mesopore generation achieved under microwave irradiation.

From a mechanistic perspective, the superior performance of the microwave-treated sample can be related to the rapid and homogeneous heating provided by microwave irradiation, which has been reported to promote more selective silicon extraction while minimizing framework damage. This results in a better preservation of the FAU structure and Brønsted acidity, along with a more uniform mesopore distribution. Such features are critical for balancing accessibility and catalytic functionality, ultimately limiting the formation of heavy aromatic precursors and suppressing coke maturation pathways. Compared to conventional hydrothermal or alkaline treatments, microwave-assisted processes have been shown to enhance textural properties while reducing defect formation, which is consistent with the lower coke condensation observed in this work. Overall, when placed in the context of the existing literature, the results obtained for Z-0.20MW demonstrate a clear advancement over both conventional microporous zeolites and standard desilicated materials. The combination of lower coke yield, reduced aromatic condensation, and enhanced coke reactivity indicates that microwave-assisted desilication provides a more effective route to control coke formation and evolution. This highlights the potential of microwave-assisted methods as a powerful tool in the design of next-generation FCC catalysts, where fine control over porosity and acidity is essential to achieve improved resistance to deactivation and enhanced regeneration performance [42].

### 2.5. Changes in Catalyst Properties After Coke Formation

Table 4 summarizes the evolution of the textural properties of the catalysts after VGOD cracking. In all cases, a decrease in BET surface area, total pore volume, and average mesopore diameter is observed, indicating progressive obstruction of the porous network by carbonaceous deposits formed during reaction. Such behavior is widely reported for zeolite-based FCC catalysts, where coke deposition reduces the accessible internal surface and limits mass transport. However, the magnitude of these changes depends strongly on both the structural features of the catalyst and the nature of the coke formed, as demonstrated in the previous FTIR and TPO analyses [43].

The parent zeolite (Z-0.00) exhibits the most severe deterioration, with a reduction in BET surface area from 696 to 472 m^2^ g^−1^ and a marked decrease in pore volume. This pronounced loss of accessible porosity is consistent with the formation of highly condensed polyaromatic coke, which tends to accumulate within the microporous FAU framework. Similar trends have been reported for conventional USY catalysts, where coke deposition within supercages and channel intersections severely restricts diffusion and blocks access to active sites. The strong confinement effects inherent to microporous structures promote the transformation of intermediate species into large polyaromatic deposits, which are difficult to remove and highly detrimental to catalyst performance [44].

The conventionally desilicated sample (Z-0.20C) exhibits an intermediate level of textural degradation. Although the introduction of mesoporosity initially improves accessibility, the data indicate that a significant fraction of this porosity becomes partially blocked during reaction, as reflected by the reduction in both surface area and average mesopore diameter. This behavior is consistent with literature reports on alkaline-treated zeolites, where the generation of mesopores is often accompanied by structural defects and extra-framework aluminum species that serve as nucleation sites for coke deposition. As a result, coke formation is not restricted to the micropores but can extend into the mesoporous network, progressively reducing its effectiveness for molecular transport.

A markedly different behavior is observed for the microwave-assisted desilicated zeolite (Z-0.20MW), which retains a substantially larger fraction of its original textural properties after reaction. The relatively small decrease in surface area and pore volume, together with the better preservation of mesopore diameter, indicates that the hierarchical structure generated under microwave conditions is more resistant to coke-induced deterioration. This behavior can be directly linked to the more controlled and homogeneous mesopore development promoted by microwave-assisted treatments, which enhance pore connectivity while preserving the crystalline framework.

In addition, microwave-assisted modification of Y zeolites has been reported to produce materials with higher mesopore surface area and improved accessibility, which translates into enhanced catalytic performance and reduced diffusion limitations for bulky molecules. Such structural features are consistent with the improved resistance to pore blockage observed for Z-0.20MW, where the more accessible and better-connected pore network facilitates the removal of coke precursors before they evolve into highly condensed species. This interpretation is fully aligned with the lower coke condensation degree evidenced by FTIR [45].

Acidity measurements further support these findings. All catalysts show a decrease in Brønsted acid sites after reaction, with the extent of loss following the order Z-0.00 > Z-0.20C > Z-0.20MW. This trend reflects the increasing severity of coke deposition and correlates with the degree of coke condensation previously discussed. Brønsted acid sites are particularly susceptible to deactivation due to their direct involvement in hydrogen-transfer and condensation reactions, as well as their interaction with nitrogen-containing compounds present in VGOD. These species can strongly adsorb or become protonated at acidic sites, leading to their blockage and reduced accessibility [46].

In contrast, Lewis acidity is less affected, showing more moderate variations after reaction. However, in materials such as Z-0.20C, where extra-framework aluminum species are more abundant, these sites can contribute to non-selective reactions that promote coke growth. The improved preservation of both Brønsted and Lewis acidity in Z-0.20MW indicates that microwave-assisted treatment not only enhances pore accessibility but also limits the formation of structural defects associated with conventional desilication. A particularly relevant observation is that the mesoporous fraction appears to be more susceptible to deterioration than the microporous fraction after reaction. This suggests that, although coke nucleation occurs primarily within micropores, the subsequent growth of carbonaceous species leads to blockage of pore mouths and micro–mesopore connectivity pathways. This phenomenon has been widely described in hierarchical zeolites, where accessibility is governed not only by pore volume but also by the connectivity between different pore systems. In this context, the improved resistance of Z-0.20MW to mesopore blockage highlights the importance of a well-connected hierarchical structure in maintaining effective molecular transport under reaction conditions.

Finally, the results demonstrate that the impact of coke deposition on catalyst properties is governed not only by the total amount of coke formed, but also by its nature and spatial distribution within the porous network. While conventional desilication improves accessibility to some extent, the associated structural degradation limits its effectiveness under severe cracking conditions. In contrast, microwave-assisted desilication enables a more controlled development of hierarchical porosity, leading to improved preservation of both textural and acidic properties after reaction. This translates into enhanced resistance to deactivation and represents a clear advancement over conventional materials, confirming the potential of microwave-assisted strategies for the design of more robust FCC catalysts.

## 3. Materials and Methods

In this study, a commercial Y zeolite was modified by alkaline desilication to generate hierarchical structures and to evaluate the influence of the treatment method on its physicochemical properties and catalytic performance. Two desilication strategies were investigated: a conventional thermal treatment and a microwave-assisted approach. The resulting materials were characterized to assess their structural, textural, and acidic properties, and subsequently evaluated in the catalytic cracking of vacuum gas oil (VGO) under microactivity test (MAT) conditions.

### 3.1. Materials

All chemical reagents were of analytical grade and used as received without further purification. Sodium hydroxide (NaOH, ≥98%), hydrofluoric acid (HF, 48 wt% in H_2_O, ≥99.99%), ammonium chloride (NH_4_Cl, ≥99.5%), hydrochloric acid (HCl, 37%), and dichloromethane (CH_2_Cl_2_, ≥99.5%) were purchased from Merck, Darmstadt, Germany. Indole (≥99%) and quinoline (≥98%), employed as model nitrogen-containing compounds for feedstock doping, were also supplied by Merck. Nitrogen with a certified purity of 99.999% was provided by Cryogas, Barranquilla, Colombia, and used as an inert carrier gas. Ultrapure water obtained from a Milli-Q Gradient A10 system (Millipore, Billerica, MA, USA) was used for all solution preparations and washing procedures. The starting catalytic material was a commercial protonated Y zeolite (CBV 760, Si/Al = 31), supplied by Zeolyst International, Conshohocken, PA, USA.

### 3.2. Feedstock Characterization

The vacuum gas oil used in this work was supplied by a Colombian refinery. The feedstock was first characterized in its original state and subsequently doped with nitrogen-containing model compounds in order to simulate high concentrations of nitrogen contaminants and to evaluate their impact on catalyst deactivation. API gravity was determined according to ASTM D287-12b [47]. The Conradson carbon residue (CCR) was measured following ASTM D4530-15 [48]. Simulated distillation curves were obtained using a Shimadzu GC-2014 gas chromatograph (Shimadzu Corporation, Kyoto, Japan) equipped with a flame ionization detector (FID) and a non-polar capillary column (30 m length, 250 μm internal diameter, and 0.25 μm film thickness), in accordance with ASTM D2887-23 [49]. The hydrocarbon fractions present in the VGOD were separated into saturates, aromatics, resins, and asphaltenes (SARA) following the ASTM D2007-11 procedure [50]. The concentrations of Ni, V, Na, and Fe were determined by inductively coupled plasma optical emission spectrometry (ICP-OES) using a PerkinElmer Optima 7300 DV instrument (PerkinElmer Inc., Waltham, MA, USA) [51].

### 3.3. Preparation and Characterization of Hierarchical Zeolites

Commercial Y zeolite was subjected to alkaline desilication using a 0.20 mol L^−1^ NaOH solution by two different approaches: conventional thermal treatment and microwave-assisted processing. After alkaline leaching, the treated samples, together with the untreated reference zeolite, were hydrothermally stabilized by steam treatment at 780 °C for 5 h. As a result, three materials were obtained: an untreated reference zeolite (Z-0.00), a conventionally desilicated zeolite (Z-0.20C), and a microwave-assisted desilicated zeolite (Z-0.20MW). Detailed experimental conditions for each technique are provided in the following sections.

#### 3.3.1. Alkaline Desilication: Conventional and Microwave-Assisted

In the conventional procedure, 5 g of zeolite were dispersed in 200 mL of 0.20 mol L^−1^ NaOH solution at 40 °C for 10 min under continuous magnetic stirring. For the microwave-assisted experiments, the same solid-to-liquid ratio and NaOH concentration were used. The resulting suspension was treated in a MARS-5 microwave reactor (CEM Corporation, Matthews, NC, USA; 2450 MHz, maximum power 1200 W), using automatic power modulation to reach the target temperature of 40 °C within approximately 2 min. The temperature was subsequently maintained at 40 °C for 10 min under continuous stirring. After alkaline desilication, both conventionally treated and microwave-treated samples were subjected to identical post-treatment procedures. All resulting suspensions were neutralized with a 0.5 mol L^−1^ HCl solution, filtered under vacuum, and thoroughly washed with deionized water. Ion exchange was subsequently carried out using a 0.50 mol L^−1^ NH_4_Cl solution at room temperature for 8 h, employing a solid-to-liquid ratio of 1 g per 10 mL of solution. The exchanged solids were then washed, filtered, and dried at 110 °C for 12 h, followed by calcination at 550 °C for 2 h with a heating rate of 10 °C min^−1^.

To evaluate their structural stability under severe FCC-like conditions, all samples were subsequently subjected to steam treatment at 780 °C for 5 h. After steaming, the samples were designated Z-0.00, Z-0.20C, and Z-0.20MW, where Z denotes zeolite, the values 0.00 and 0.20 correspond to the NaOH concentration used during alkaline treatment, and the letters C and MW indicate the desilication method (conventional or microwave-assisted), respectively. The efficiency of the desilication process was calculated as the ratio between the mass of zeolite recovered after calcination and the initial mass subjected to alkaline treatment. Depending on the treatment method, desilication yields ranged between 80% and 90%.

#### 3.3.2. Zeolite Characterization

The obtained zeolites were characterized to evaluate the structural modifications induced by the desilication treatments. Crystallographic properties were investigated by powder X-ray diffraction (XRD) using a Siemens D500 diffractometer (Siemens AG, Karlsruhe, Germany) equipped with CuKα monochromatic radiation (λ = 1.5418 Å). Diffraction patterns were collected over the 2θ range of 5–50°, using a step size of 0.05° and a counting time of 3 s per step. The relative crystallinity of the samples was determined from the diffraction patterns, while the framework Si/Al ratio was estimated according to the Breck–Flanigen correlation, providing an indirect assessment of the extent of framework silicon extraction after alkaline treatment [52].

Textural properties were determined by nitrogen physisorption at 77 K using a Micromeritics 3Flex™ automatic analyzer (Micromeritics Instrument Corporation, Norcross, GA, USA) [53]. Prior to analysis, the samples were degassed under vacuum (10^−6^ mmHg) at 300 °C for 24 h. Nitrogen adsorption–desorption isotherms were recorded at −196 °C. The specific surface area was calculated using the Brunauer–Emmett–Teller (BET) method, whereas the total pore volume was estimated from the amount of nitrogen adsorbed at a relative pressure (P/P_0_) of approximately 0.98. Micropore volume and external surface area were determined by the t-plot method, while mesopore size distribution and average pore diameter were obtained using the Barrett–Joyner–Halenda (BJH) model.

The acidic properties of the fresh and coked zeolites, including the density, nature, and strength of the acid sites, were characterized by Fourier transform infrared (FTIR) spectroscopy of adsorbed pyridine (Py-FTIR) and pyridine temperature-programmed desorption (Py-TPD) [54]. FTIR spectra were recorded on a Nicolet spectrophotometer using a spectral resolution of 4 cm^−1^ and 256 accumulated scans. Self-supported wafers (15 mm diameter) were prepared by pressing 80 mg of zeolite under a pressure of 4 tons cm^−2^. Prior to pyridine adsorption, the samples were degassed at 450 °C for 2 h under a vacuum of 10^−3^ Torr, cooled to room temperature, and the background spectrum was subsequently recorded. Pyridine (Merck, 99.5 wt%) was adsorbed at 150 °C, and spectra were collected in the 1700–1400 cm^−1^ region after desorption at 150, 300, and 400 °C, allowing differentiation of acid sites according to their thermal stability and acid strength. The absorption bands centered at 1540 and 1450 cm^−1^ were assigned to pyridinium ions associated with Brønsted acid sites and coordinatively bonded pyridine on Lewis acid sites, respectively, following the assignments proposed by Emeis [55]. The concentrations of Brønsted and Lewis acid sites were calculated from the integrated absorbance of the corresponding bands using the molar extinction coefficients reported by Emeis [55], namely 1.67 cm μmol^−1^ for the Brønsted band (1540 cm^−1^) and 2.22 cm μmol^−1^ for the Lewis band (1450 cm^−1^). These coefficients are widely accepted for the quantitative determination of acid site densities in zeolitic materials and enable direct comparison with previous studies.

The acid site density and strength were further evaluated by pyridine temperature-programmed desorption (Py-TPD). Before pyridine adsorption, the samples were pretreated under flowing nitrogen at 400 °C for 1 h. Pyridine was subsequently adsorbed from a saturator at 150 °C for 60 min, followed by purging with nitrogen at the same temperature for 1 h to remove physisorbed molecules. The samples were then heated from 150 to 780 °C at a rate of 15 °C min^−1^, and the desorbed pyridine was analyzed by gas chromatography using a flame ionization detector (FID) with a Shimadzu GC-2014 gas chromatograph (Shimadzu Corporation, Kyoto, Japan).

### 3.4. Catalytic Evaluation Tests

The catalytic performance of the three Y zeolites, both treated and untreated, was evaluated by means of microactivity tests conducted in a fixed-bed Microactivity Test (MAT) reactor, following well-established protocols designed to simulate fluid catalytic cracking (FCC) conditions at laboratory scale. This experimental approach allows a reliable assessment of the intrinsic catalytic behavior of FCC-type materials under controlled and reproducible conditions. The experiments were carried out using a Colombian vacuum gas oil (VGO), previously characterized and subsequently doped in the laboratory with nitrogen-containing model compounds, namely quinoline and indole, in order to simulate feedstocks with different levels and chemical natures of nitrogen contamination. These compounds were selected for their representative basic and non-basic nitrogen functionalities commonly found in heavy refinery streams. For each catalytic run, 3 g of catalyst were loaded into the reactor, while the VGOD was fed at a mass flow rate of 2.0 g·min^−1^. The reactions were performed at a constant temperature of 550 °C, with contact times ranging from 20 to 50 s. These operating conditions were selected to ensure that variations in conversion, product selectivity, and coke formation could be directly attributed to differences in catalyst structure and feed composition. All experiments were conducted under continuous nitrogen flow, which was used as carrier gas.

The reaction products were collected and analyzed by gas chromatography. Catalytic performance was evaluated in terms of VGO conversion and product selectivities toward dry gas, LPG, gasoline, and coke. Conversion (X), product selectivities (*Sj*), and coke selectivity (*Scoke*) were calculated according to Equations (1)–(3):

Equation (1). Feed conversion

The feed conversion (wt%) was calculated as the sum of the yields of all reaction products according to:

X (%) = Y_DG_ + Y_LPG_ + Y_GASOLINE_ +Y_COKE_

Equation (2). Product selectivity

The selectivity (wt%) toward product j was calculated as the ratio between the yield of product j and the overall feed conversion:

$$S_{j}=\frac{Y_{j}}{X}100$$

where j corresponds to dry gas (DG), liquefied petroleum gas (LPG), gasoline, and coke.

Equation (3). Product yield

The yield (wt%) of each product j was calculated from the mass of product recovered after reaction according to:

$$Y_{j}=\frac{m_{j}}{m_{rec}}100$$

where m*j* is the mass of product j and m*_rec_* is the mass of feed introduced into the reactor.

All experiments were performed at least in duplicate to ensure the reproducibility and reliability of the experimental results.

#### Coke Characterization

The amount of coke deposited on the zeolites after the catalytic tests was first quantified by mass balance. Subsequently, the nature and combustion behavior of the deposited coke were investigated by temperature-programmed oxidation (TPO), a technique widely used to assess coke reactivity and its implications for catalyst regeneration in FCC processes. TPO experiments were carried out to obtain combustion profiles of the coked zeolites, providing insight into the strength of coke–catalyst interactions and the relative ease of coke removal. During the oxidation process, the carbon oxides (CO and CO_2_) formed were converted into methane over a nickel catalyst in the presence of hydrogen and quantified using a flame ionization detector (FID). The resulting oxidation profiles enabled differentiation between coke species with distinct thermal stabilities and oxidation behaviors.

Additional information on the chemical nature of the deposited coke was obtained from the background FTIR spectra of the coked samples. In particular, absorption bands centered at approximately 1580 cm^−1^ were assigned to aromatic carbonaceous species, while bands around 1610 cm^−1^ were associated with olefinic coke structures. These spectral features allowed a qualitative comparison of coke composition among the different catalysts. To further investigate the composition of the soluble fraction of coke, the coked zeolites were subjected to a selective dissolution of the zeolitic framework using hydrofluoric acid (48 wt.% in H_2_O, ≥99.99%), thereby releasing the carbonaceous deposits retained within the porous structure. The extracted coke species were subsequently recovered by liquid–liquid extraction using dichloromethane (ACS reagent, ≥99.5%). The resulting soluble coke fraction was then analyzed by gas chromatography to gain additional insight into its molecular composition. This extraction methodology is a well-established approach for isolating and characterizing coke species occluded within zeolitic materials.

## 4. Conclusions

This work provides a comprehensive evaluation of the effect of conventional and microwave-assisted desilication on the catalytic performance of Y zeolites in the cracking of vacuum gas oil (VGOD), with particular emphasis on coke formation, coke nature, and catalyst stability. The results clearly demonstrate that catalyst performance is governed by a subtle interplay between porosity, acidity, and mass transport properties, which ultimately controls both the formation and the evolution of carbonaceous deposits.

The parent zeolite (Z-0.00), characterized by a purely microporous structure and a high density of strong Brønsted acid sites, exhibited the highest coke yields and the most severe deactivation. Coke formed over this material was predominantly composed of highly condensed polyaromatic species, as evidenced by FTIR and TPO analyses, and was preferentially located within the microporous network. This resulted in extensive pore blockage, significant loss of accessible surface area, and a substantial decrease in acidic functionality after reaction.

The introduction of hierarchical porosity through conventional alkaline desilication (Z-0.20C) partially mitigated these limitations by improving mass transport and reducing coke formation. However, this treatment also induced structural defects and generated extra-framework aluminum species, which promoted non-selective reaction pathways and sustained the formation of relatively condensed coke. As a result, Z-0.20C exhibited intermediate behavior, where improved accessibility was partially offset by increased structural heterogeneity and limited resistance to coke-induced deterioration.

In contrast, the microwave-assisted desilicated zeolite (Z-0.20MW) consistently showed superior performance across all evaluated aspects. This catalyst exhibited the lowest coke yield, the lowest degree of coke condensation, and the highest resistance to deactivation. The coke formed over Z-0.20MW was predominantly composed of less condensed, more reactive species, which were more readily oxidized during TPO analysis. This behavior is attributed to the more homogeneous and well-connected mesoporous network generated under microwave conditions, which enhances molecular transport, reduces the residence time of reactive intermediates, and suppresses secondary reactions leading to coke maturation.

Importantly, the improved coke characteristics directly translated into better preservation of catalyst properties after reaction. Z-0.20MW retained a larger fraction of its surface area, pore volume, and Brønsted acidity compared to both the parent and conventionally treated zeolites. This highlights that catalyst stability is not solely determined by the amount of coke formed, but also by its chemical nature and spatial distribution within the pore network.

Overall, this study demonstrates that microwave-assisted desilication provides a more effective strategy than conventional alkaline treatment for the generation of hierarchical Y zeolites with enhanced resistance to coke formation and deactivation. The ability to simultaneously control porosity, preserve structural integrity, and tailor acidity results in catalysts that are better suited for processing heavy feeds under severe FCC conditions. These findings not only advance the understanding of coke formation and catalyst deactivation in hierarchical zeolites, but also highlight the potential of microwave-assisted approaches for the rational design of next-generation FCC catalysts with improved stability and regeneration performance. Future work should focus on scaling microwave-assisted treatments and evaluating catalyst performance under industrial FCC conditions to further validate their practical applicability.

## Figures and Tables

**Table 1:** Physicochemical and elemental properties of the VGOs.

Feedstock	VGO	VGOD
°API	18.5	18.1
Aniline point (°C)	76.9	74.8
CCR (wt%)	0.46	0.50
Refractive index	1.493	1.504
Distillation curve (°C)		
Initial	270	277
10 vol.%	376	381
30 vol.%	415	422
50 vol.%	446	453
70 vol.%	481	490
95 vol.%	529	537
Final	575	582
SARA fractions (wt%)		
Saturated	49.1	46.1
Aromatic	48.5	51.1
Resin	1.90	2.10
Asphaltene	0.50	0.70
Nickel (ppm)	0.55	0.56
Vanadium (ppm)	0.86	0.85
Sodium (ppm)	0.91	0.89
Iron (ppm)	0.30	0.27
Quinoline (wt%)	-	0.40
Indol (wt%)	-	0.40
Nitrogen Total (wt%)	0.10	0.90

**Table 2:** Textural, structural, and acidic properties of the parent and desilicated Y zeolites.

Textural Properties	Z-0.00	Z-0.20C	Z-0.20MW
BET specific surface area, *S_BET_* (m^2^/g)	696	584	625
Mesopore-specific surface area, *S_meso_* (m^2^/g)	98	166	238
Total pore volume, *V_TP_* (cm^3^/g)	0.446	0.510	0.543
Micropore volume, *V_micro_* (cm^3^/g)	0.342	0.234	0.195
Mesopore volume, *V_meso_* (cm^3^/g)	0.104	0.276	0.348
Average mesopore diameter, ${\bar{d}}_{p}$ (Å)	50.2	66.5	88.4
**Crystalline properties**			
Crystallinity (%)	100	64	79
Unit cell size (Å)	24.28	24.31	24.33
Si/Al in the framework	31.0	27.0	28.0
**Acidic properties**			
Brønsted acidity			
Total (μmol Py/g)	186	158	172
Strong (%)	40	32	36
Lewis acidity			
Total (μmol Py/g)	69	82	74
Strong (%)	31	36	33

**Table 3:** Product distribution (selectivities, wt%) and gasoline composition for VGOD conversion at 550 °C (72 wt% conversion).

Selectivities (wt%)	Z-0.00	Z-0.20C	Z-0.20MW
S_DG_	16.2	11.6	7.10
S_LPG_	19.5	20.4	21.5
S_GASOLINE_	52.0	58.5	63.0
***Gasoline composition (%)***			
Paraffins	25.2	19.7	16.0
Olefins	28.5	32.4	35.5
Naphthenes	21.9	20.6	18.3
Aromatics	24.4	27.3	30.2
RON	84.0	87.2	89.7
S_COKE_ (wt%)	12.8	9.5	6.6

**Table 4:** Textural and acidic properties of the parent and modified fresh and coked zeolites used in the cracking of VGOD (72 wt% conversion).

	Z-0.00		Z-0.20C		Z-0.20MW	
Properties	Fresh	Coked	Fresh	Coked	Fresh	Coked
BET specific surface area, *S_BET_* (m^2^/g)	696	472	584	425	625	529
Total pore volume, *V_TP_* (cm^3^/g)	0.446	0.263	0.510	0.352	0.543	0.488
Average mesopore diameter, ${\bar{d}}_{p}$ (Å)	50.2	30.1	66.5	44.1	88.4	70.9
Brønsted acidity						
Total (μmol Py/g)	186	119	158	116	172	141
Lewis acidity						
Total (μmol Py/g)	69	40	82	62	74	62

## Data Availability

The data presented in this study are available in this article.
